# Advancing the science and practice of effective mentorship

**DOI:** 10.1017/cts.2025.10168

**Published:** 2025-10-28

**Authors:** Christine Pfund, Christine Sorkness, David Asai, Marcus Lambert, Emma Anne Meagher, Audrey J. Murrell, Nancy Schwartz, Joel Tsevat

**Affiliations:** 1 Institute for Clinical and Translational Research (ICTR) and Center for the Improvement of Mentored Experiences in Research (CIMER), University of Wisconsin-Madison, Madison, WI, USA; 2 School of Pharmacy and Institute for Clinical and Translational Research (ICTR), University of Wisconsin-Madison, Madison, WI, USA; 3 Howard Hughes Medical Institute (formerly), Chevy Chase, MD, USA; 4 SUNY Downstate Health Sciences University, Brooklyn, NY, USA; 5 Perelman School of Medicine, University of Pennsylvania, Philadelphia, PA, USA; 6 School of Business, School of Public and International Affairs, Department of Psychology, Clinical and Translational Science, University of Pittsburgh, Pittsburgh, PA, USA; 7 Department of Pediatrics and Biochemistry and Molecular Biology, Division of Biological Sciences, University of Chicago, Chicago, IL, USA; 8 Center for Research to Advance Community Health and Division of General Internal Medicine, Department of Medicine, Long School of Medicine, University of Texas Health Science Center at San Antonio, San Antonio, TX, USA

**Keywords:** Mentorship, career development, workforce development, peer mentoring, assessment

## Introduction

In 2019, the National Academies of Sciences, Engineering, and Medicine published a consensus study report on the science and practice of effective mentorship. This report outlined the current state of knowledge on mentorship, identified gaps in knowledge, and offered recommendations for advancing a culture of mentorship within institutions [1]. Since then, the study of mentorship has continued to grow at a rapid pace, with significant work being done in the context of translational science. This special issue provides an opportunity to document some of this progress and identify emerging themes for future studies.

There is a rich history of innovations in mentorship in translational science. Improving the quality of mentoring practice has been a core mission of Clinical and Translational Science Award (CTSA) programs since their inception. Since the beginning, the National Institutes of Health (NIH) CTSA application criteria have required programs to describe how mentors of career development award scholars and training grant trainees are trained and evaluated. One of the earliest CTSA committees focused on mentorship. That group included the Research Education and Career Development Award Directors from 18 CTSA hubs who collectively published a series of papers focused on the various elements of mentorship [2–8]. In addition, the first randomized controlled trial of a mentor training intervention was conducted with mentors and mentees from 16 CTSA sites and expanded to additional CTSA hubs from there [9,10]. Many leaders of the NIH Diversity Program Consortium-funded National Research Mentoring Network (NRMN) are based at CTSAs [11]. In 2022, the National Center for Advancing Translational Sciences (NCATS) supported a national conference on the science and practice of mentorship, which sparked the development of a mentorship community of practice [12].

This thematic issue of JCTS includes 15 papers selected from peer-reviewed manuscripts, which aligned with the themes described in an open call for submissions. These papers further deepen understanding of effective mentorship and how findings are being translated into the practices of individual mentors and academic institution ecosystems. The papers cover a range of topics, including new mentorship training models and career development programs in biomedical research aimed at improving mentoring relationships across diverse contexts. The first article in this thematic issue by Ianni and colleagues provides a scoping review of mentorship studies in the CTSA context [13]. The remaining 14 articles describe exciting approaches to advancing the science and practice of mentorship across the clinical and translational science landscape.

## Emerging themes and future research directions

From the collection of papers in this issue, we noted four emerging themes, each pointing to future studies.

### Workforce development skill building through mentorship interventions

This *JCTS* thematic issue includes four articles describing interventions focused on workforce development skill building in the areas of mentorship, uncovering the hidden curriculum (implicit norms and behaviors), scientific communication, and grantsmanship. First, in their special communication, K. Cameron and co-authors describe a faculty mentor training workshop series and certificate program. Participants in the program reported improved mentoring skills and a parallel increase in frequency of recommended mentoring behaviors [14]. Enders and colleagues [15] describe a conceptual framework and competencies that can help career development award scholars to more effectively identify and navigate the hidden curriculum in academia. C. Cameron and co-authors [16] share results from a novel communication intervention supported by NRMN aimed at improving the ability of participants to develop communication skills between mentors and mentees across a variety of characteristics in clinical and translational research settings. Finally, in their brief report, Hernandez and team share results from participants in two activities: a professional ecosystem mapping activity and a mentor-mentee similarity activity [17]. Taken together, the frameworks and interventions described are ready for further testing to identify what works, for whom, and in what context.

### The power of peer/near-peer mentoring

Four special communications and two research articles describe the development, assessment, and study of innovative peer/near-peer mentoring programs. Each approach has elements that can inform ways to support early career investigators in clinical and translational science by using peer/near-peer mentoring program designs. For example, Griendling and co-workers [18] report on a 9-month mentoring program for early career faculty, post-doctoral fellows, research scientists, and clinical fellows with their mentors by utilizing effective one-on-one mentoring and peer mentoring activities. In their special communication about the Health Equity Leadership and Mentoring Program, Steiner and team describe a new cohort-based mentoring model for early-stage investigators committed to careers in health equity research and clinical care. Early evaluation results indicate the program enhances psychosocial safety and feelings of belonging [19]. Lovinsky-Desir and co-authors share details about their peer/near-peer ASPIRE! program and how their approach can enhance the experience of early-career research faculty [20]. Palmer and colleagues [21] explored how group coaching and mentoring can lead to enhanced self-reflection, sense of community, shared experience, and increased empowerment. Pololi and team share encouraging results from their C-Change Mentoring and Leadership Institute, a group peer mentoring program focusing on leadership development and structured career planning for mid-career faculty in academic medicine [22]. Finally, an important element of peer/near-peer mentoring programs is the facilitation of peer-to-peer interactions and the management of group dynamics among members of each mentoring cohort. Strekalova and team [23] report results of a qualitative study of grantsmanship coaches in one of the NRMN-based programs.

Collectively, these articles highlight the power of peer/near-peer mentoring and the careful attention in terms of structure and facilitation required for such approaches to be effective, especially when implemented in groups. The results also illustrate the importance of more research on the impact of peer/near-peer support on the experiences of early career academicians.

### Assessment and comparative effectiveness of mentorship experiences

Advancing the science of mentorship requires assessing influencing factors and outcomes. Here, using valid measures is key, and using common measures across time or different groups facilitates comparisons. In this issue, Rogers and Byars-Winston [24] describe the development and testing of a 12-item measure to assess interpersonal dynamics within mentoring relationships. Their scale helps pave the way for studies of how mentorship interventions influence mentorship working alliances between mentors and mentees and enhance the overall quality of mentoring experiences. Waugh and researchers report their comparison of mentored research experiences of undergraduates historically not included in science and medicine across differing institutional contexts. Their study emphasizes the need for future research to better understand the influences of science identity and scientific roles on the experiences of developing researchers [25].

### Dissemination and implementation

A core principle of translational science is the ability to move research into practice efficiently [26]. The same is true of promising workforce development interventions. In their paper, Weber-Main and co-authors [27] evaluated an online mentor training module with a large sample of users from hundreds of institutions (mainly in CTSA hub training programs). Results indicate this module can serve as an effective, standalone, asynchronous approach for mentor development or as one curricular component of a comprehensive, multimodal program. Using a dissemination and implementation framework, Spencer and co-workers [28] report on factors such as leadership support that promote and limit implementation of mentorship education interventions and suggest areas for both additional focus and further study.

## Conclusion

Several of the programs and studies reported in this thematic issue took place in the context of CTSAs and included scholars engaged in translational science. Others describe interventions and findings that can be applied to clinical and translational science settings. Overall, this assembly of articles presents exciting frameworks, promising interventions, and ideas for future research that are critical to advancing the science and practice of mentorship. What has been learned so far and will be learned in the future can contribute to programmatic and institutional culture changes aimed at optimizing the experiences of mentors and mentees working together to advance translational science.
